# The methylomes of six bacteria

**DOI:** 10.1093/nar/gks891

**Published:** 2012-10-02

**Authors:** Iain A. Murray, Tyson A. Clark, Richard D. Morgan, Matthew Boitano, Brian P. Anton, Khai Luong, Alexey Fomenkov, Stephen W. Turner, Jonas Korlach, Richard J. Roberts

**Affiliations:** ^1^New England Biolabs, 240 County Road, Ipswich, MA 01938 and ^2^Pacific Biosciences, 1380 Willow Road, Menlo Park, CA 94025, USA

## Abstract

Six bacterial genomes, *Geobacter metallireducens* GS-15, *Chromohalobacter salexigens*, *Vibrio breoganii* 1C-10, *Bacillus cereus* ATCC 10987, *Campylobacter jejuni* subsp. jejuni 81-176 and *C. jejuni* NCTC 11168, all of which had previously been sequenced using other platforms were re-sequenced using single-molecule, real-time (SMRT) sequencing specifically to analyze their methylomes. In every case a number of new *N*^6^-methyladenine (^m6^A) and *N*^4^-methylcytosine (^m4^C) methylation patterns were discovered and the DNA methyltransferases (MTases) responsible for those methylation patterns were assigned. In 15 cases, it was possible to match MTase genes with MTase recognition sequences without further sub-cloning. Two Type I restriction systems required sub-cloning to differentiate their recognition sequences, while four MTase genes that were not expressed in the native organism were sub-cloned to test for viability and recognition sequences. Two of these proved active. No attempt was made to detect 5-methylcytosine (^m5^C) recognition motifs from the SMRT® sequencing data because this modification produces weaker signals using current methods. However, all predicted ^m6^A and ^m4^C MTases were detected unambiguously. This study shows that the addition of SMRT sequencing to traditional sequencing approaches gives a wealth of useful functional information about a genome showing not only which MTase genes are active but also revealing their recognition sequences.

## INTRODUCTION

We are becoming accustomed to the ever-increasing speed and reduced cost with which DNA can be sequenced. However, what is often lost in this frenzy of sequencing is the fact that DNA consists of more than just four bases. In eukaryotes, we have known for a long time about the epigenetic role of 5-methylcytosine (^m5^C), sometimes called the fifth base, and more recently it has been found that 5-hydroxymethylcytosine, 5-formylcytosine and 5-carboxylcytosine are also present (1–4). However, two more modified bases, *N*^6^-methyladenine (^m6^A) and *N*^4^-methylcytosine (^m4^C), are also common in bacterial genomes, where they function as components of restriction–modification (RM) systems (5). Until recently, these have usually been ignored because of the lack of simple methods to determine their locations. However, with the advent of single-molecule, real-time (SMRT) sequencing (6–8), it has suddenly become possible to detect these modified bases as a part of the routine sequencing procedure.

The methylated bases that are found in bacterial and archaeal genomes serve important functions as part of RM systems, where they protect the host chromosome against the otherwise deleterious action of the partner restriction enzyme(s), which are needed to destroy unwanted incoming transmissible DNA elements such as phages (9). However, in some cases these methyltransferases (MTases) also serve regulatory roles as with the Dam MTase of *Escherichia coli*, which introduces ^m6^A residues that play a key role in DNA repair and also have important effects during the initiation of replication (10). Several studies have also implicated MTases in regulating gene expression, phase variation and pathogenicity (11,12). Given the many DNA MTases that are typically found in prokaryotic genomes, it seems likely that they will have hitherto undocumented effects aside from their key role in RM systems. To date, there has been no genome-wide assessment of the extent of DNA methylation by known MTases such as *E. coli* Dam (10) and Dcm (13) or the cell cycle MTase, CcrM, of *Caulobacter crescentus* (14). It is not known if their methylation specificities are as precise as the customary recognition sequences suggest or whether the enzymes are promiscuous. This is particularly interesting to know for RM systems as there are no obvious selective constraints on MTase specificity provided that the core recognition sequence of the restriction enzyme is fully modified.

Recently, we have shown that by cloning an individual MTase gene into a plasmid and propagating it in an otherwise methylation-deficient strain of *E. coli*, it is easily possible through SMRT sequencing to detect all of the bases modified on the plasmid (15). Precise recognition sequences were convincingly demonstrated and mostly matched that of the cognate restriction enzyme when the MTase was part of an RM system. However, some promiscuous methylation was observed, with the Dam gene of *E. coli* being a particularly striking example. There was one caveat to this interpretation though: because the MTase genes in that study were cloned on a multi-copy number plasmid (50–200 copies per cell), it could be that the observed promiscuity arose because of over-expression.

Given that the results for the plasmids were very clear, it seemed that it might be possible to perform a direct analysis of bacterial genomes using the SMRTsequencing method and thus obtain an accurate estimate of the extent of methylation in the native organism. By then, comparing a bioinformatic analysis of the RM systems with the direct measurement of just what was methylated, it should be possible to assign recognition sequences to individual MTase genes. Of particular interest in this sort of analysis are the Type I and Type III RM systems, which have generally been very difficult to analyze by previous, more tedious techniques (16). In both of these kinds of systems, the specificity comes from a single subunit of the enzyme—the S subunit of the Type I enzymes and the M subunit of the Type III enzymes (16). Thus, it seemed likely that recognition sequences for both types of MTases could be discovered relatively easily. To demonstrate the feasibility of this approach, we chose initially to analyze six genomes with relatively few RM systems before moving on to more complicated cases.

## MATERIALS AND METHODS

### Materials

All restriction endonucleases (REases) except Eco147I (Fermentas; Glen Burnie, MD, USA), Phusion-HF DNA polymerase, Antarctic Phosphatase, T4-DNA ligase and *E. coli* competent cells were from New England Biolabs Inc. (Ipswich, MA, USA). Synthetic oligonucleotides were purchased from Integrated DNA Technologies (Coralville, IA, USA). *Geobacter metallireducens* GS-15 ATCC 53774 DNA, *Chromohalobacter salexigens* DSM 3043 DNA and *Bacillus cereus* ATCC 10987 DNA were obtained from the culture collections indicated. *Vibrio breoganii* 1C-10 DNA was a gift from Martin Polz, MIT. *Campylobacter jejuni* subsp. jejuni 81-176 and *C. jejuni* NCTC 11168 DNAs were a gift from Stuart Thompson, Medical College of Georgia.

### SMRT sequencing

SMRTbell template libraries were prepared as previously described (15,17). Briefly, genomic DNA samples were sheared to an average size of ∼800 bp via adaptive focused acoustics (Covaris; Woburn, MA, USA), end repaired and ligated to hairpin adapters. Incompletely formed SMRTbell templates were digested with a combination of Exonuclease III (New England Biolabs; Ipswich, MA, USA) and Exonuclease VII (Affymetrix; Cleveland, OH, USA). SMRT sequencing was carried out on the PacBioRS (Pacific Biosciences; Menlo Park, CA, USA) using standard protocols for small insert SMRTbell libraries. Sequencing reads were processed and mapped to the respective reference sequences using the BLASR mapper (http://www.pacbiodevnet.com/SMRT-Analysis/Algorithms/BLASR) and the Pacific Biosciences' SMRTAnalysis pipeline (http://www.pacbiodevnet.com/SMRT-Analysis/Software/SMRT-Pipe) using the standard mapping protocol. Interpulse durations were measured as previously described (7) and processed as described (15) for all pulses aligned to each position in the reference sequence. To identify modified positions, we used Pacific Biosciences' SMRTPortal analysis platform, v. 1.3.1, which uses an *in silico* kinetic reference and a *t*-test based kinetic score detection of modified base positions (details are available at http://www.pacb.com/pdf/TN_Detecting_DNA_Base_Modifications.pdf).

MTase target sequence motifs were identified by selecting the top 1000 kinetic hits and subjecting a ±20 base window around the detected base to MEME-ChIP (18). To measure the extent of methylation for each motif in a genome, a kinetic score threshold was chosen such that 1% of the detected signals were not assigned to any MTase recognition motifs (5% for *B. cereus* to accommodate for the lower signal intensities for ^m4^C). We subjected this 1% population of sequence context to another round of MEME-ChIP analysis to confirm the absence of any additional consensus motifs. We observed no accumulation of motifs that harbored similarities to the identified active motifs. All kinetic data files have been deposited in GEO (accession numbers GSE40133) (19) (http://www.ncbi.nlm.nih.gov/geo/summary/).

### Bioinformatic analysis

The SEQWARE computer resource was used to identify RM system genes from the complete genome sequences of *G. metallireducens* GS-15 (GenBank numbers CP000148 and CP000149), *C. salexigens* (GenBank number CP000285), *B. cereus* (GenBank numbers AE017194 and AE017195), *C. jejuni* subsp. jejuni 81-176 (GenBank numbers CP000538, CP000549 and CP000550), *C. jejuni* NCTC 11168 (GenBank number AL111168) and *V. breoganii* 1C-10 (GenBank number AKXW00000000). Software modules combined with internal databases constitute the SEQWARE resource. New sequence data are scanned locally for homologs of already identified and annotated RM systems in REBASE (5). Sequence similarity from BLAST searches, the presence of predictive functional motifs (20,21) and genomic context are the basic indicators of potential new RM system components. Heuristic rules, derived from knowledge about the gene structure of RM systems, are also applied to refine the hits. Attempts are made to avoid false hits caused by strong sequence similarity of RNA and protein MTases or hits based solely on non-specific domains of RM enzymes, such as helicase or chromatin remodeling domains. SEQWARE then localizes motifs and domains, assigns probable recognition specificities, classifies accepted hits and marks Pfam relationships. All candidates are then inspected manually before being assigned as part of an RM system. The results are entered into REBASE (5).

### MTase cloning

Selected MTase genes were amplified from bacterial genomic DNA with Phusion-HF DNA polymerase and cloned into the plasmid pRRS as described previously (15). Gene-specific oligonucleotide primers used for PCR are described in Supplementary Table S1. When no suitable sites were present elsewhere in the construct, restriction sites diagnostic for the predicted methylation pattern were incorporated into the 3′-end oligonucleotides. The presence or absence of specific methylation was determined by digesting the constructs with appropriate restriction enzymes. Host strains used for cloning included *E. coli* ER2796 (22) and *E. coli* ER2683 (23).

The Csa_1401 and Gmet_0255 genes were cloned into the plasmid pRRS using the Gibson assembly technique (24). The pRRS vector was PCR amplified using primers pRRS srbs for and pRRS rev. The MTase genes were amplified using primers having 5′ tails that overlap with the ends of the amplified pRRS vector (Supplementary Table S1). PCR amplified DNAs were purified over a Qiagen spin column. A total of 0.1 pmol vector was combined with 0.3 pmol MTase gene insert in 20 µl 1× Gibson assembly reaction (New England Biolabs) and incubated at 50°C for 1 h. A total of 2 µl of this assembled construct was transformed into 50 µl chemical competent *E. coli* ER2796 cells and plated on LB-ampicillin plates at 37°C overnight.

## RESULTS

We analyzed six bacterial strains, all of which had relatively few predicted RM systems and several of which had some experimental data already available. Three of these strains, *G. metallireducens* GS-15, *C. salexigens* and *V. breoganii* 1C-10 had never been tested for active MTases previously, while three other strains, *B. cereus* ATCC 10987, *C. jejuni* subsp. jejuni 81-176 and *C. jejuni* NCTC 11168 were all known to contain several active MTases (25–27). In each case there were Type I or Type III RM systems for which no information was available about either their activity or recognition sequences.

We analyzed each genome using SEQWARE and made predictions about the RM systems that were present including REase and MTase genes and recognition sequences when a gene showed high similarity to a biochemically characterized gene of known recognition sequence. These predictions are summarized for all RM system components in Supplementary Table S2. Each genome was then subjected to SMRT sequencing and the methylated bases identified by their kinetic signatures (7). These were then aligned and clustered to identify the motifs that constituted the consensus recognition sequences for the MTases. These experimental results were then matched with the bioinformatic predictions. Several factors helped in this matching such as the fact that all known Type III MTases and most Type IIG systems only methylate one strand of their recognition sequence. Type I systems have bipartite recognition sequences in which two short motifs (3–5 nt long) are separated by 5–8 non-specific nucleotides. A well-known example is the EcoKI RM system that recognizes 5′-A^m6^ACNNNNNNGTGC-3′ (28). Methylation takes place as indicated (T indicates that the A residue on the complementary strand is methylated). It should be noted that because ^m5^C generates a weak and somewhat diffuse SMRTsignal (7) no attempt was made in any of these whole genome analyses to identify the position of ^m5^C in the complete genome analyses. Rather, where appropriate these MTase genes were cloned and analyzed separately as was done previously (15).

### *Geobacter metallireducens* GS-15

**Figure 1.**
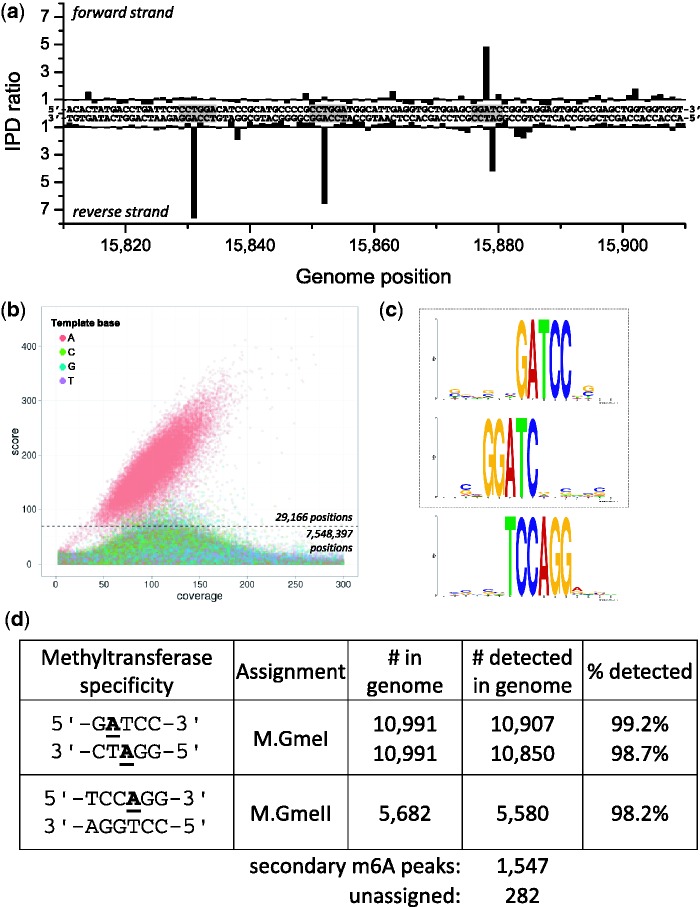
Methylome determination of *G. metallireducens* GS-15. (**a**) Example trace of kinetic variation, showing three instances of methylated sequence regions. (**b**) Scatter plot of sequencing coverage and kinetic score for all genomic positions. The colors indicate the bases as shown in the upper left of the panel. The cutoff for detected genomic positions is indicated by the dashed line. (**c**) MTase specificities determined from the genomic positions detected as methylated. They are highlighted as gray boxes in the example trace (a). (**d**) Summary of detected methylated positions across the genome.

*Geobacter metallireducens* strain GS-15, first isolated from freshwater sediment, is capable of reducing iron, manganese, uranium and other metals and thus represents an interesting target for bioremediation of groundwater contaminants (29). The genome sequence of this organism, which grows at 30°C, was originally determined by the Joint Genome Institute (JGI) (GenBank numbers CP000148 and CP000149). Bioinformatic analysis indicated that there should be two MTases associated with Type II RM systems and one with a Type III system (Supplementary Table S2). Two active MTases were detected based on the SMRT sequencing analysis (Figure 1; Supplementary Figures S1a and S2a). Figure 1a shows kinetic signals for both DNA strands for a section of the genome containing three instances of detected regions containing methylated template bases, two of which are limited to one of the two DNA strands and the other encompassing methylation on both DNA strands. Genome-wide analysis of all template positions (Figure 1b) revealed a population of A bases that clearly separated from the background of all other template positions. Motif analysis (see ‘Materials and Methods’ section) resulted in the identification of two MTase specificities: 5′-G^m6^ATCC-3′ and 5′-TCC^m6^AGG-3′ (Figure 1c). The extent of methylation across the genome was determined by considering 29 166 positions detected as methylated, corresponding to >99% of all hits matching a motif (Figure 1b; see ‘Materials and Methods’ section). Greater than 98% of all genomic positions matching these MTase specificities were detected as methylated (Figure 1d).

Of the two Type II systems, one (Gmet_3140) showed great similarity to known MTases recognizing 5′-GGATC-3′, including M.EacI (30) and M.AlwI (5). In all cases, the MTase is itself a fusion of two MTase domains, one recognizing 5′-GGATC-3′ and forming 5′-GG^m6^ATC-3′ and the other recognizing the complementary strand and forming 5′-G^m6^ATCC-3′. The new MTase identified here is called M.GmeI and its corresponding REase encoded by Gmet_3138 is called GmeIP, since it is not known if it is active. Interestingly, Gmet_3138 shows great similarity to the known restriction enzyme genes EacI (30) and AlwI (5), but unlike the latter two genes, which are immediately adjacent to their respective MTase genes, the genes for M.GmeI and GmeIP are separated by an open reading frame encoding a protein of 333 amino acids, which is homologous to a protein in the same location in *G. metallireducens* RCH3, but has much less similarity to other proteins in GenBank. However, the next closest homolog is a 509 amino acid protein in *Syntrophothermus lipocalidus* DSM 12680, which also sits next to an MTase gene, but one of different recognition specificity (5′-ACCTGC-3′).

The other Type II MTase (Gmet_0255) contained the typical motifs associated with an ^m5^C DNA MTase, but its recognition sequence could not be predicted as the variable region showed no great similarity to other ^m5^C MTases of known specificity. This MTase was cloned and tested for its ability to incorporate ^3^H-methyl groups into DNA using labeled *S*-adenosylmethionine as substrate, but was found to be inactive. Similarly, no DNA methylation was observed by SMRT sequencing of the plasmid containing the cloned gene (data not shown). Either this MTase is inactive or it could be an RNA MTase.

The Type III MTase (Gmet_0676) clearly recognizes 5′-TCC^m6^AGG-3′ and modifies the A residue as indicated. It is named M.GmeII. As with all known Type III enzymes, only one strand is modified. It too has a corresponding REase gene as the adjacent ORF (Gmet_0675), but it is not known if it is active.

During our analysis, we found that there appeared to be a deletion in the genomic DNA we obtained from the ATCC relative to the reference genome, as we observed no sequencing coverage between positions 2 446 610 and 2 588 100. This region is flanked by two transposase genes. This deletion has also been observed by Dr Derek Lovley (unpublished data).

### Chromohalobacter salexigens

*Chromohalobacter salexigens* is a moderate halophile that is tolerant to various salt environments and allows other organisms (e.g. Salmonella) to exist in environments they would otherwise not be able to cope with. The genome sequence of this organism, which grows at 37°C, was originally determined by the JGI (31). Bioinformatic analysis of the genome indicated that there should be one Type I system and two Type II systems (Supplementary Table S2). The recognition sequence of the Type I system could not be predicted since the specificity subunit (Csal_0086), which determines the recognition sequence, showed no similarity to any well-characterized system. Of the Type II systems, one (Csal_1368) was predicted to recognize 5′-GATC-3′ since it showed significant similarity to several well-characterized 5′-GATC-3′ MTases. However, the recognition sequence of the second Type II MTase (Csal_1401), which appears to be encoded on a prophage, could not be predicted. It was suspected that this might not be active in the genome as frequently prophage-encoded genes are transcriptionally inactive until such time as the prophage is excised (32).

**Figure 2.**
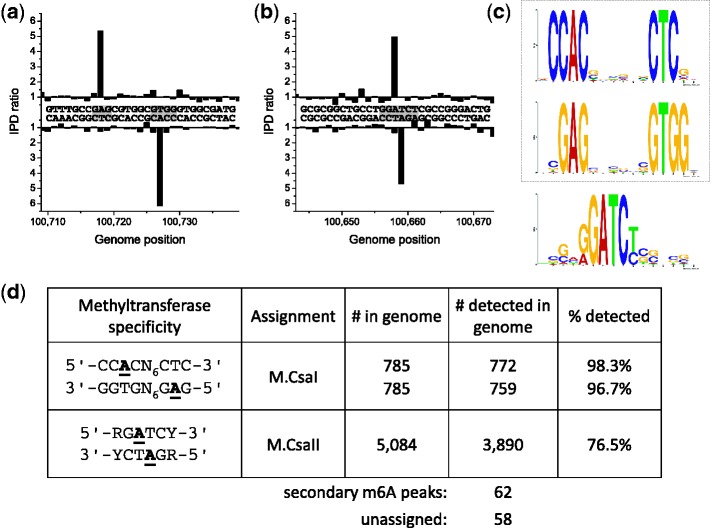
Methylome determination of *C. salexigens*. (**a** and **b**) Example traces of kinetic variation, showing two instances of methylated positions. (**c**) MTase specificities determined from the genomic positions detected as methylated. (**d**) Summary of detected methylated positions across the genome.

The results of whole genome SMRT sequencing analysis are shown in Figure 2 and demonstrate that the putative GATC MTase is expressed, methylates the adenine residues on both strands to form ^m6^A, but actually recognizes the more specific sequence, 5′-RGATCY-3′, although methylation seems not to be complete during normal growth. This MTase is called M.CsaI. The specificity was very strict as the number of hits observed for 5′-NGATCN-3′, but not conforming to 5′-RGATCY-3′, was 0 (Supplementary Figure S3). The Type I system is very well defined and recognizes the usual bipartite sequence pattern recognized by Type I enzymes, but this particular recognition sequence 5′-CCAC(N)_6_CTC-3′ has not been reported previously (5). As usual for Type I systems, the MTase, M.CsaII, acts on the single adenine residue in each DNA strand forming ^m6^A. The putative prophage-encoded MTase appears not to be expressed. That the 5′-RG^m6^ATCY-3′ signal is due to expression of Csal_1368 and is not a combination of expression of both Type II ORFs was tested by cloning Csal_1401 separately in the methylation deficient *E. coli* strain ER2796 (22). The resulting clone showed that the MTase was non-specific and methylated most, but not all, A residues in the plasmid (Supplementary Figure S4). Motif analysis indicated the following specificity rules for this relatively non-specific MTase: 5′-^m6^AB-3′ and 5′-S^m6^AAM-3′ (>96% of all hits with a kinetic score >100 fell into these motifs; B = not A; S = G or C, M = A or C).

### *Vibrio breoganii* 1C-10

**Figure 3.**
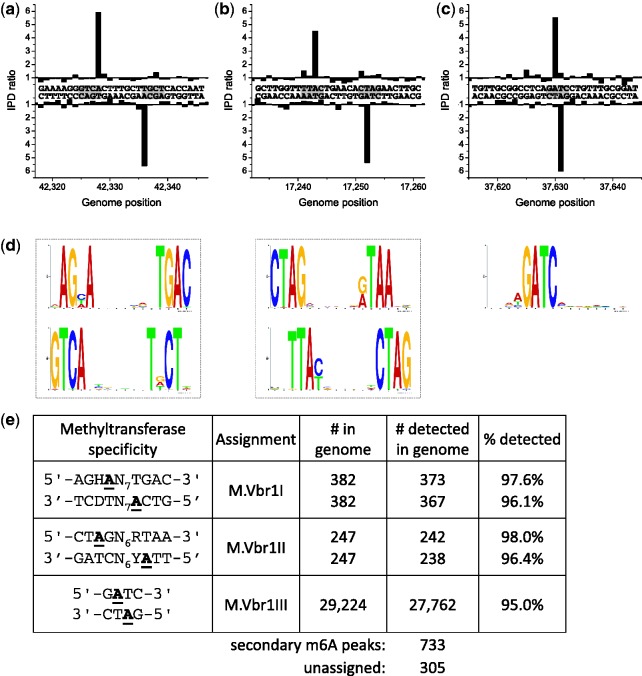
Methylome determination of *V. breoganii* 1C-10. (**a–c**) Example traces of kinetic variation, showing instances of the detected methylated motifs. (**d**) MTase specificities determined from the genomic positions detected as methylated. (**e**) Summary of detected methylated positions across the genome.

*Vibrio breoganii* is a non-motile, alginolytic, marine bacterium. Strain 1C-10 was isolated from large suspended particles (likely macroalgal detritus) during analysis of resource partitioning of *Vibrionaceae* populations (33,34). Bioinformatic analysis suggested that this strain contained two Type I RM systems and both proved to be active, methylating the sequence motifs 5′-AGH^m6^A(N)_7_TGAC-3′ and 5′-CT^m6^AG(N)_6_RTAA-3′, respectively (Figure 3; Supplementary Figures S1c and S2c). Bioinformatics alone could not resolve which system recognized which sequence and so the M and adjacent S genes of the two systems were cloned as pairs. The S1.VbrIP gene is about half the length of a typical S subunit and was not tested for activity. The resulting plasmids tested for resistance to HindIII and ScaI to test for methylation by M.VbrI and M.VbrII, respectively (Supplementary Figure S5). The partial protection against HindIII is expected for an MTase, M.VbrI, forming 5′-AGC^m6^AAGCTTAATGAC-3′ as the resulting hemi-methylated HindIII site does not completely inhibit cleavage (5). In a parallel experiment, methylation by M.VbrII gave complete protection against ScaI at the sequence 5′-CT^m6^AGTACTCCATA-3′ as expected (5). These assignments were confirmed by SMRT sequencing of the plasmids containing individual MTase-expressing clones (Supplementary Figure S6).

Again from bioinformatic analysis, there were two Type II MTases present. The first, M.VbrDam, was a close homolog of the M.EcoKDam MTase of *E. coli* (35) and indeed the genome was methylated at essentially all GATC sites as predicted (Figure 3). The second MTase was enigmatic and while a very weak signal (192 out of the 305 unassigned hits) that could be interpreted as C^m4^CA was found by sequencing, this seemed unlikely to be the recognition sequence since very few genomic positions harboring this motif had strong kinetic signals. Consistent with this hypothesis, no modified sites were detected upon cloning this gene into a plasmid and analysis by SMRT sequencing (data not shown), indicating that this MTase gene is inactive. The weak CCA signals are more likely the result of phosphorothioated nucleotides which have been detected in this bacterium by bulk methods [(36); T. A. Clark and J. Korlach, unpublished data].

### *Campylobacter jejuni* subsp. jejuni 81-176

**Figure 4.**
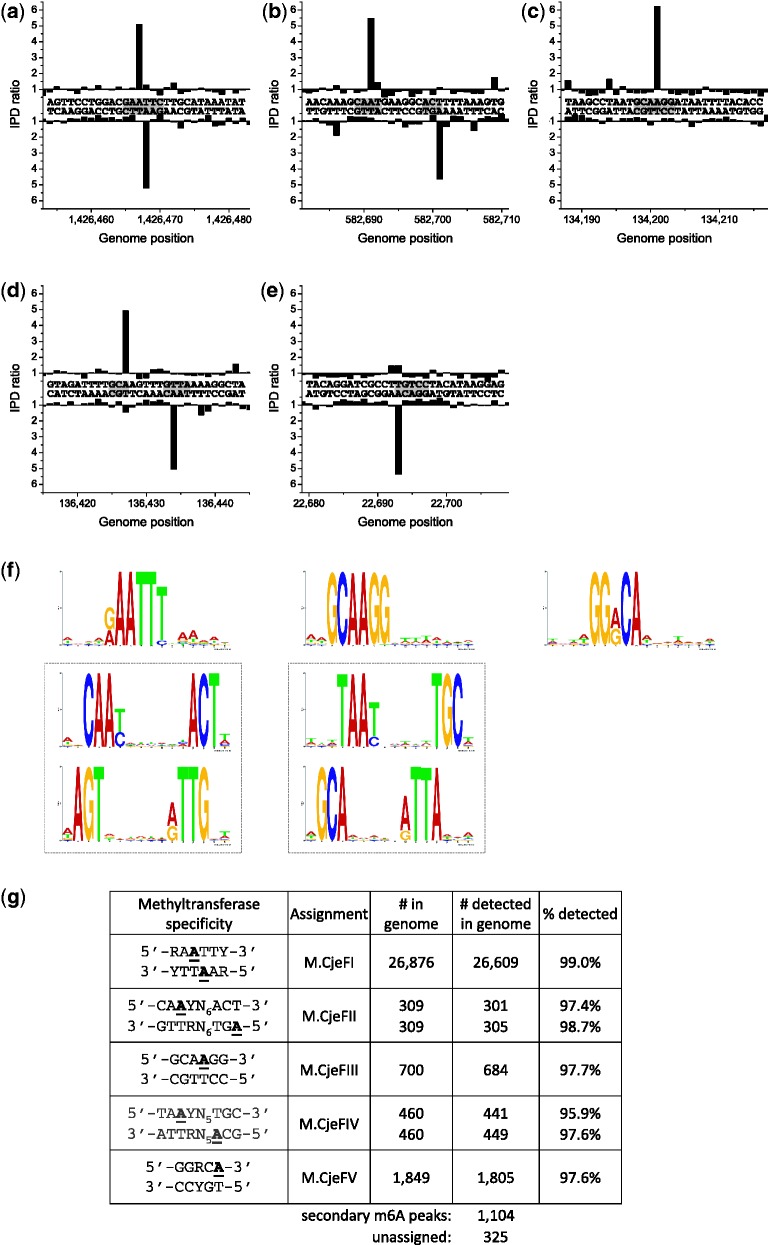
Methylome determination of *C. jejuni* 81-176. (**a–e**) Example traces of kinetic variation, showing instances of the detected methylated motifs. (**f**) MTase specificities determined from the genomic positions detected as methylated. (**g**) Summary of detected methylated positions across the genome.

**Figure 5.**
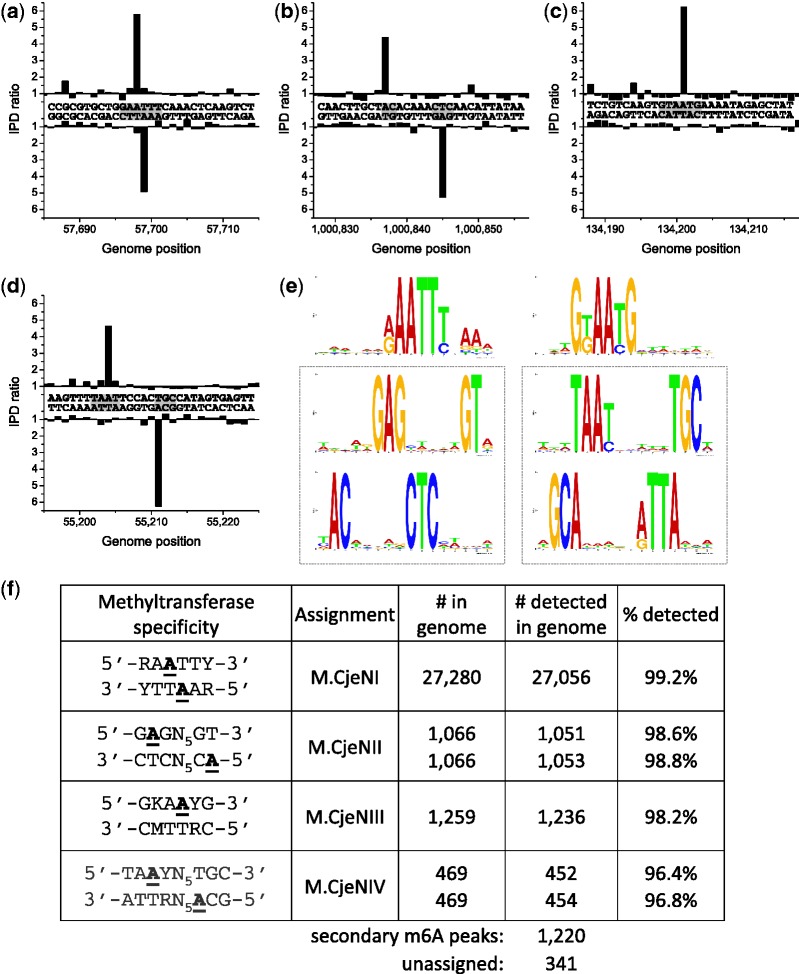
Methylome determination of *C. jejuni* NCTC 11168. (**a–d**) Example traces of kinetic variation, showing instances of the detected methylated motifs. (**e**) MTase specificities determined from the genomic positions detected as methylated. (**f**) Summary of detected methylated positions across the genome.

*Campylobacter jejuni* is a Gram-negative bacterium native to the digestive tract of poultry and other bird species and is one of the most common causes of human gastroenteritis. The genome sequence of this organism had been determined some time ago (D. Fouts and K. Nelson, unpublished data; GenBank numbers CP000538, CP000549 and CP000550). Bioinformatic analysis suggested the presence of two Type I RM systems and four Type II systems, several of which had close homologs in *C. jejuni* NCTC 11168 (Supplementary Table S2). One gene, CJJ81176_0240, was 99% identical to the characterized gene for M.CjeNI, which was reported to recognize 5′-GAATTC-3′ (26). However, when examining the genomic methylation through SMRT sequencing, it was clear that the gene in this strain, coding for M.CjeFI, recognized the more degenerate sequence 5′-RA^m6^ATTY-3′ (Figure 4); the same proved true for M.CjeNI (see below and Figure 5). Another MTase gene, CJJ81176_1454, was extremely similar to a gene in *C. jejuni* NCTC 11168 that was reported to encode an active 5′-GATC-3′ MTase (27). However, in neither of the two *Campylobacter* strains was such an active MTase detected. Furthermore, the gene in question shows more similarity to the RNA MTase RsmD than to other DNA MTases. We conclude that this gene is not able to methylate DNA and its true activity may require further biochemical investigation. Two additional MTase genes appear to be part of Type IIG RM systems in which sequence specificity, methylation and restriction are all carried out by the same polypeptide. One recognizes the sequence 5′-GGRCA-3′ and modifies the terminal A residue, while the other recognizes the sequence 5′-GCAAGG-3′ and modifies the second A residue (Figure 4). As with many other Type IIG enzymes, only one strand of the DNA is methylated. To decide which gene was which, we noticed that CJJ81176_0713 is very similar to Cj0690c in *C. jejuni* NCTC 11168, which recognizes the related sequence 5′-GKAAYG-3′ (see below). Thus, we assigned CJJ81176_0713 as the gene encoding RM.CjeFIII forming 5′-GCA^m6^AGG-3′ and CJJ81176_0068 as the gene encoding RM.CjeFV forming 5′-GGRC^m6^A-3′ (Figure 5). These assignments were confirmed by cloning the individual ORFs and testing the clones for protection from appropriate REases (Supplementary Figure S7). These results are summarized in Table 1.

**Table 1.** Bioinformatic predictions and experimental results for all MTase genes

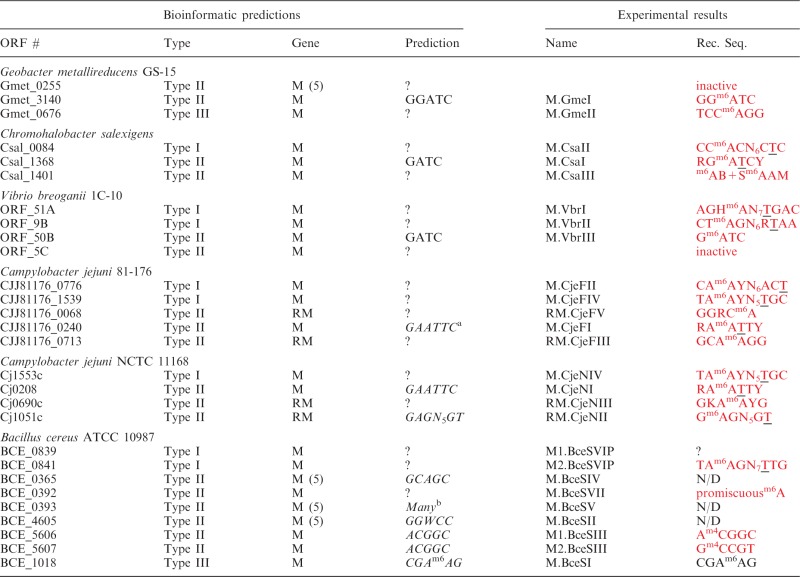

Italicized genes characterized previously; red text indicates new information or revision. Recognition sequences representations use the standard abbreviations. (*Eur. J. Biochem.*, **150**, 1–5, 1985) to represent ambiguity: R = G or A, Y = C or T, M = A or C, K = G or T, S = G or C, W = A or T, = not A (C or G or T), D = not C (A or G or T), H = not G (A or C or T), V = not T (A or C or G), N = A or C or G or T.

N/D = not detected (^m5^C assignments were not attempted).

^a^indicates incorrect result obtained previously.

^b^5′-GGCC-3′/5′-GCNGC-3′/5′-CCGG-3′/5′-GGNCC-3′ are all recognized.

Finally, the two Type I systems are both active with one forming 5′-CA^m6^AYN_6_ACT-3′ and the other forming 5′-TA^m6^AYN_5_TGC-3′. Since only the second of these modifications is present in *C. jejuni* NCTC 11168, it can be safely concluded that the specificity subunit, CJJ81176_1536, which has a close homolog in that strain, recognizes 5′-TAAYN_5_TGC-3′ and the specificity subunit, CJJ81176_0777, recognizes 5′-CAAYN_6_ACT-3′. In both cases, methylation results in the second A residue being modified as shown in Figure 4.

### *Campylobacter jejuni* NCTC 11168

This strain (37) codes for one Type I RM system and four Type II systems. The Type I system is essentially identical with the CjeFIV system in *C. jejuni* subsp. jejuni 81-176 and forms 5′-TA^m6^AYN_5_TGC-3′ (CjeNIV) (Figure 5). Two of the Type II systems, M.CjeNI and RM.CjeNII, had previously been characterized [26; J.M.B. Vitor *et al.*, unpublished data (5)]. However, as noted earlier, M.CjeNI recognizes 5′-RAATTY-3′ (Figure 5) rather than 5′-GAATTC-3′ as had been reported (26). RM.CjeNII is a Type IIG system and recognizes 5′-GAGN_5_GT-3′ and is now shown to methylate both A residues on the two strands. Another Type II MTase is encoded by Cj0690c and is a Type IIG enzyme that forms 5′-GKA^m6^AYG-3′ methylating the second A residue (Figure 5). This gene was cloned in *E. coli* and found to produce active endonuclease recognizing 5′-GKAAYG-3′ and cutting 19/17 downstream. From the bioinformatic analysis, one additional gene, Cj0031, plus the adjacent gene, Cj0032, looks like a Type IIG enzyme containing a frameshift. The complete gene would be 99% identical to the gene for RM.CjeFV, which recognizes 5′-GGRCA-3′. However, no such modification is found in the genome confirming that the frameshift is real and that this frameshifted gene produces no active MTase. SMRT sequencing data confirmed the presence of the frameshift.

### *Bacillus cereus* ATCC 10987

This bacterium was originally isolated from spoiled cheese and belongs to the same genetic subgroup as *Bacillus anthracis* (38). The RM systems in *B. cereus* ATCC 10987 had previously been examined by Xu *et al.* (25), who determined recognition sequences for four Type II and III REases and one orphan MTase by traditional methods. However, the sites of methylation for the Type II and III MTases were not determined and several other MTases were not examined including that in the Type I system (BCE_0839-BCE_0842) and a Type II MTase (BCE_0392) that was reported to be inactive (25). However, when we cloned this MTase and checked its activity, it was clearly a promiscuous ^m6^A MTase, which we have now named M.BceSVII (Supplementary Figure S9 and Table 1).

**Figure 6.**
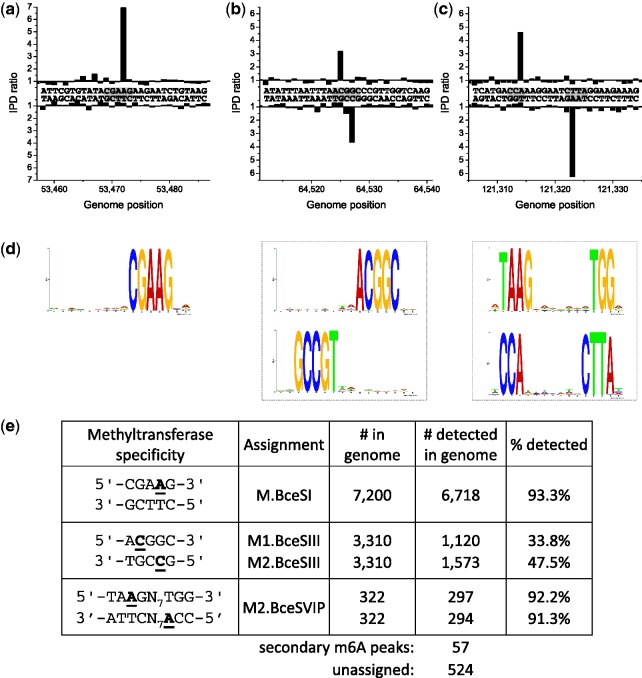
Methylome determination of *B. cereus* ATCC 10987. (**a–c**) Example traces of kinetic variation, showing instances of the detected methylated motifs. (**d**) MTase specificities determined from the genomic positions detected as methylated. (**e**) Summary of detected methylated positions across the genome.

Our main goal was to characterize the Type I system and also ascertain the sites of methylation by the MTases not addressed in the previous study. The Type I system, now called BceSVI, was clearly active and recognized the sequence 5′-TA^m6^AGN_7_TGG-3′, where again the underlined T indicates ^m6^A on the complementary strand (Figure 6; Supplementary Figures S1f and S2f). This system is a little unusual in that, it contains two M subunits. Because we did not clone the individual components of this system, we cannot say whether one or both M subunits are active. The sites of modification of the three other Type II MTases are indicated in Table 1, while the Type III MTase, which had been identified earlier by cloning, is shown to be completely active in the genome.

The previously identified Type II REase BceSIII recognizes an asymmetric sequence, 5′-ACGGC-3′ and requires two MTases for protection, both of which are ^m4^C MTases. These form 5′-A^m4^CGGC-3′ in the strand shown and 5′-G^m4^CCGT-3′ in the complementary strand (Figure 6b). To show which MTase recognizes which strand, we cloned the two MTase genes independently and checked for their ability to protect against appropriate REases (Supplementary Figure S8). From this analysis, we can conclude that M1.BceSIII forms 5′-A^m4^CGGC-3′ and M2.BceSIII forms 5′-G^m4^CCGT-3′. It is important to note that while cloning the individual MTase genes showed five to be active only four seem to be active in the genome. M.BceSV, a multi-specific MTase characterized in the previous study by cloning and overexpression (25) is encoded on a prophage and does not show detectable activity in the native host genome. In addition to the ^m6^A and ^m4^C MTases mentioned earlier, our analysis indicated two more motifs that are likely modified by one or more of the predicted ^m5^C MTases in the *B. cereus* genome, as 179 of the 524 unassigned hits fell into two categories. These motifs were 5′-G^m5^CWGC-3′ and 5′-GGWC^m5^C-3′ which are consistent with recognition specificity predictions for BCE_0365 and BCE_4605 (Supplementary Table S2). The kinetic signals for ^m5^C are subtle in that with the kinetic score cutoff used, we detect only 138 5′-GCWGC-3′ (out of 15416 in the genome) and 41 5′-GGWCC-3′ (out of 5460) sites. We are currently exploring methods of enhancing the kinetic signature of ^m5^C during SMRT sequencing.

## DISCUSSION

The results presented in this article and summarized in Table 1 represent one of the first times that it has been possible to examine the complete methylation pattern of a bacterial genome. For the MTases studied in this article, seven are components of Type I RM systems and have six different recognition sequences, all of which are new. Two Type III systems were found with one new recognition sequence. Two MTases were part of traditional Type II systems although we did not test whether the REase was active. Four Type IIG REases, which contain both MTase and REase activity in a single polypeptide chain, were found, all with new specificities. It should be noted that two of these, RM.CjeFIII and RM.CjeNIII, show very high sequence similarity and yet recognize different sequences (5′-GCA^m6^AGG-3′ and 5′-GKA^m6^AYG-3′, respectively). Thus, this finding represents another family of Type IIG restriction enzymes that resemble the MmeI family, where a few simple changes in critical base recognition elements cause changes in specificity (39). This again emphasizes the need for caution when transferring annotation from one characterized protein to another (40). The composition of an amino acid change can be critical if it occurs at a residue belonging to a DNA sequence recognition element. Two orphan MTases, M.CsaIII and M.BceSVII, were found to be active when cloned, but inactive in the genome. Both are promiscuous ^m6^A MTases and both occur on prophage elements suggesting that they may play a protective role during phage infection. Finally, two solitary 5′-GATC-3′ MTases were shown to be active. It should be noted that when examining complete genome sequences for MTases, some of the genes may be inactive because of mutation, while others may be inactive due to transcriptional silencing as is often found when the genes are present as part of a prophage. In the latter case cloning can reveal methylation activity, permitting complete characterization as found earlier (15).

One of the striking features of the results from the current analysis is that the recognition sequences of all MTases found to be active showed fairly strict specificity with very few off-target events noted. Of course, much greater coverage would be required to detect very rare off-site effects and so some degree of promiscuity cannot be ruled out. However, the apparent promiscuity that was observed in our earlier work (15) using MTase genes cloned in high copy number plasmids was not apparent. We consider the ‘true’ MTases specificity to be reflected in the modification patterns seen when they are expressed in their genomic context. Thus, based on the current findings, we would have to conclude that in general it seems likely that most MTases show essentially identical specificity to their cognate REases, a result that was not completely expected since there are no obvious constraints on their specificity.

Previously, it had been found that Type III MTases only methylate a single strand of their recognition sequence and that holds true here. Similarly, most characterized Type IIG enzymes methylate just a single strand although several do not, including RM.CjeNII as described here. Nevertheless, this can be very helpful when trying to match recognition sequences found by sequencing with the genes responsible for each consensus sequence. Another useful feature is that all known Type I restriction systems seem to possess split recognition sequences, which can help in distinguishing them when matching genes and consensus sequences. Nevertheless, if two Type I systems are present as in *V. breoganii* 1C-10, it was essential to clone out the individual systems so that specificity and genes could be properly matched. Note that because of the mechanism of methylation it is only the M and S subunits that need to be cloned to permit assembly of a functional MTase (16).

In the case of the Type II RM system BceSIII, because of the asymmetric nature of the recognition sequence, two independent MTases are required to methylate each strand of the sequence. While SMRT sequencing can easily find the locations of each methyl group, it was necessary to clone out the two MTase genes separately in order to assign strand specificity to each one. M.GmeI also recognizes an asymmetric sequence, but in this case, the two M genes are fused. At the present time, we have relatively little information about strand specificity of MTases, because it has proven difficult to determine specificity experimentally. As more data accumulate using the kinds of analyses that we present here, it should become much easier in the future to make accurate bioinformatic predictions about recognition sequences and specificity for MTases in newly sequenced genomes.

Despite the recognized importance of methylation for understanding fundamental microbiological processes, microbe adaptability and disease pathogenicity (11,12), in the past, there has not been a great deal of research into the methylation patterns of bacterial genomes, largely because of the difficulty of obtaining suitable data. One area where knowledge about the methylome is very important relates to studies trying to transform DNA into strains that contain one or more RM systems and which vastly reduce transformation efficiencies. In some cases, these barriers have been overcome by premethylating the DNA or by removing the RM systems from strains (41,42). One problem with the latter approach is that removal of methylation systems may fundamentally change the biology of the organism under study. With the kind of analysis provided here, the RM systems likely to cause problems with transformation can be easily spotted and appropriate measures taken. Thus, the MTases necessary for protection can be identified and if needed intermediate cloning hosts carrying suitable complements of MTase genes can be prepared.

In summary, the results provided here show that SMRT sequencing can provide functional information about active MTases present in genomes and can decipher their recognition sequences, a task that used to be time-consuming to a point where it was not usually carried out. This, combined with the long reads provided by this technology can be an excellent adjunct to current high-throughput sequencing platforms, in that sequence assembly is facilitated and gene function is reliably documented.

## SUPPLEMENTARY DATA

Supplementary Data are available at NAR Online: Supplementary Tables 1 and 2 and Supplementary Figures 1–9.

## FUNDING

New England Biolabs; Pacific BioSciences and NIH grants [1RC2GM092602 to J.K. and R44 GM100560 to R.J.R.]. Funding for open access charge: New England Biolabs’ internal funds.

*Conflict of interest statement*. T.A.C., M.B., K.L., S.T. and J.K. are full-time employees at Pacific Biosciences, a company commercializing SMRT sequencing technologies. I.A.M., R.D.M., A.F., B.P.A. and R.J.R. are full-time employees of New England Biolabs, a company that sells research reagents such as DNA MTases.

## Supplementary Material

Supplementary Data

Supplementary Data
