# Supplementary material for: The methylomes of six bacteria
Source: Nucleic Acids Res. 2012 Oct 2;40(22):11450–62. doi: 10.1093/nar/gks891 (PMC3526280; doi:10.1093/nar/gks891)
Supplement: Supplementary Data [file supp_40_22_11450__index.html]

The methylomes of six bacteria — The methylomes of six bacteria — Supplementary Data 

# The methylomes of six bacteria

## Supplementary Data

files

**Files in this Data Supplement:**

- Supplementary Data - pdf file
