# Supplementary material for: The methylomes of six bacteria
Source: Nucleic Acids Res. 2012 Oct 2;40(22):11450–62. doi: 10.1093/nar/gks891 (PMC3526280; doi:10.1093/nar/gks891)
Supplement: Supplementary Data [file supp_gks891_nar-01944-h-2012-File002.pdf]

Supplementary Information

Supplementary Figure 1 Scatter plot of coverage and kinetic score for (a) *Geobacter metallireducens* GS-15, (b) *Chromohalobacter salexigens*, (c) *Vibrio breoganii* 1C-10, (d) *Campylobacter jejuni* 81-176, (e) *Campylobacter jejuni* NCTC11168, and (f) *Bacillus cereus* ATCC 10987. The cutoff chosen for detected methylated genomic positions is indicated by the dashed line.

Supplementary Figure 2 Kinetic score distributions for (a) *Geobacter metallireducens* GS-15, (b) *Chromohalobacter salexigens*, (c) *Vibrio breoganii* 1C-10, (d) *Campylobacter jejuni* 81-176, (e) *Campylobacter jejuni* NCTC11168, and (f) *Bacillus cereus* ATCC 10987. The cutoff chosen for detected methylated genomic positions is indicated by the dashed line.

Supplementary Figure 3 Specificity assignment of M.CsaI in *Chromohalobacter salexigens* as 5'-RG^{m6}A$\underline{T}$CY-3', rather than the predicted specificity of 5'-G^{m6}A$\underline{T}$C-3'. (a) Example section of the genome with three instances of 5'-GATC-3' not conforming to 5'-RGATCY-3' (grey boxes), showing lack of kinetic signal. (b) Comparison of kinetic signals for the targeted 5'-RGATCY-3'motif vs. all other 5'-NGATCN-3' motifs not conforming to the target motif.

Supplementary Figure 4 Specificity assignment of M.CsaIII. The gene encoding M.CsaIII was cloned into pRRS, expressed in *E. coli* ER2796 and analyzed for methylation as described previously (15). (a) Circos plot of IPD ratios across the entire plasmid. The outer and inner circles denote the forward and reverse DNA strands, respectively. (b) IPD ratio plot for a section of the plasmid. Motif analysis indicated the following specificity rules for this relatively non-specific MTase: 5'-^{m6}AB-3', and 5'-S^{m6}AAM-3' (>96% of all hits with a kinetic score > 100 fell into these motifs; B=not A; S=G or C, M=A or C).

Supplementary Figure 5 Specificity assignment of *Vibrio breoganii* 1C-10 Type I enzymes.

M+S2.VbrI and M+S.VbrII genes were amplified by PCR using oligonucleotide primers (Supplementary Table 1), subcloned into the pRRS plasmid vector using unique PstI and BamHI restriction sites, and propagated in *E. coli* ER2796 to yield plasmid DNAs that should be devoid of methylated bases other than those arising from the activities of the cloned Type I genes. PCR primers were designed to incorporate the Type I recognition sites previously identified by SMRT sequencing of *V. breoganii* 1C-10 genomic DNA, specifically 5'-AGH^{m6}AN$_7$TGAC-3' and 5'-CT^{m6}AGN$_6$RTAA-3', adjacent to the termination codon of the relevant S subunits. Cleavage sites for HindIII (5'-AAGCTT-3') or ScaI (5'-AGTACT-3') restriction endonucleases were included within the Type I recognition sites so that the N6-modified adenine base in the top strand corresponds to the 5' nucleotide of the HindIII or ScaI site. As these nucleases are each blocked by N^6-modification of the 5'-adenine they can be used as reporters of methylation activity of the cloned Type I genes. Unmethylated control DNAs were synthesized by PCR amplification of the complete plasmid molecules by inverse PCR using oligonucleotide primers that prime synthesis at a site immediately adjacent to the unique PstI site within the vector DNA, as described previously (15). Plasmid and control DNAs were restricted by PstI and either HindIII or ScaI and the products analyzed by TBE/agarose gel electrophoresis.

Lane 1: PCR-amplified pRRS: M+S2.VbrI DNA restricted with PstI and HinDIII.
Lane 2: pRRS: M+S2.VbrI DNA restricted with PstI and HindIII.
Lane 3: PCR-amplified pRRS: M+2.VbrII DNA restricted with PstI and ScaI.
Lane 4: pRRS: M+S.VbrII DNA restricted with PstI and ScaII.

Marker = NEB 1kb-ladder (10.0, 8.0, 6.0, 5.0, 4.0, 3.0, 2.0, 1.5, 1.0 and 0.5 kb, respectively)

Supplementary Figure 6 Specificity assignments of (a) M.VbrI and (b) M.VbrII. The genes encoding for the two Type I systems were cloned into pRRS, expressed in *E. coli* ER2796 and analyzed for methylation as described previously (15). The top panels show circos plots of IPD ratios across the entire plasmid. The outer and inner circles denote the forward and reverse DNA strands, respectively. The bottom panels show IPD ratio plots for a section of the plasmid.

Supplementary Figure 7 Restriction digests confirming the specificities of M.CjeFI, RM.CjeFIII and RM.CjeFV. Plasmid constructs containing these genes (purified from *E. coli* ER2683 and linearized with PstI, or amplified by inverse PCR at the PstI site to create methylation-free controls) were digested with diagnostic REases and products resolved on an agarose gel. Lanes 1-3, 7-8, and 11-13 are linearized constructs from *E. coli*; lanes 4-6, 9-10, and 14-16 are unmethylated controls. Lanes 1-6, CJJ81176_0240 (M.CjeFI), demonstrate substantially complete protection from EcoRI (5'-GAATTC-3'; lanes 2 and 5) and ApoI (5'-RAATTY-3'; lanes 3 and 6) cleavage. Lanes 7-10, CJJ81176_0068 (RM.CjeFV) demonstrate protection from SphI (5'-GCATGC-3'; lanes 8 and 10) cleavage where it overlaps the RM.CjeFV site (5'-GGGCA-3'), but not at a second SphI site. Lanes 11-16, CJJ81176_0713 (RM.CjeFIII), demonstrate partial protection from Eco147I (5'-AGGCCT-3'; lanes 12 and 15) and nearly complete protection from StuI (5'-AGGCCT-3'; lanes 13 and 16) cleavage. Two inverted RM.CjeFIII sites were engineered such that a central StuI site would be methylated on both strands by RM.CjeFIII: 5'-GCA^{m6}AGGCC$\underline{T}$TGC-3'. Also indicated on the gel are 1 kb ladder size standards.

Supplementary Figure 8 Restriction digests confirming the specificities of M1.BceSIII and M2.BceSIII. Both genes were cloned in two versions, with differing diagnostic sites downstream of the stop codon: 5'-CGTACGGC-3' (BceSIII overlapping BsiWI and RsaI) or 5'-ACGGCGCC-3' (BceSIII overlapping KasI). Plasmid constructs (purified from *E. coli* ER2796 and linearized with PstI, or amplified by inverse PCR at the PstI site to create methylation-free controls) were digested with diagnostic REases and products resolved on an agarose gel. Top-strand methylation (5'-A^{m4}CGGC-3') should protect against BsiWI and RsaI, but not KasI, and bottom-strand methylation (5'-ACG$\underline{G}$C-3') should protect against KasI but not BsiWI or RsaI.

Lanes 1-4, KasI digestion of constructs containing the KasI-BceSIII overlapping sites, show that the M2.BceSIII-methylated construct alone is protected from KasI cleavage. Lanes 5-8, RsaI digestion of constructs containing the RsaI-BceSIII overlapping sites, show that the M1.BceSIII-methylated construct alone is protected from RsaI cleavage. Lanes 1 and 5, M1.BceSIII construct from *E. coli*; lanes 2 and 6, M1.BceSIII construct unmethylated control; lanes 3 and 7, M2.BceSIII construct from *E. coli*; lanes 4 and 8, M2.BceSIII construct unmethylated control. Fragments protected by methylation are indicated by red asterisks. Also indicated on the gel are 1 kb, 2-log, and 100 bp ladder size standards. Digests with NarI were consistent with KasI, and digests with BsiWI were consistent with RsaI (data not shown).

Supplementary Figure 9 Specificity assignment of M.BceSVII. The gene encoding M.BceSVII was cloned into pRRS, expressed in *E. coli* ER2796 and analyzed for methylation as described previously (15). (a) Circos plot of IPD ratios across the entire plasmid. The outer and inner circles denote the forward and reverse DNA strands, respectively. (b) IPD ratio plot for a section of the plasmid. Motif analysis indicated this was a relatively non-specific ^{m6}A MTase.

Supplementary Table 1. Sequences of primers used to clone MTase genes[a.]

M.GmeORF255 for	5'-TGCCTGCAGTTAAGGTTTAACATATGCAGGTCAAGGAAGGAGAGG-3'
M.GmeORF255 rev	5'-TCTAGATCTTCCCCGGGGATCCTTATCACGGCGACAATCCCAGAGA-3'
M.CsaIII for	5'-TGCCTGCAGTTAAGGTTTAACATATGACCATGAAACCGATCCTCCCAT-3'
M.CsaIII rev	5'-TCTAGATCTTCCCCGGGGATCCTTATCAGCGGTTGGTGATGATCAGCT-3'
RM.CjeNIII for	5'-GTTCTGCAGTTAAGGTTTAACATATGAATTACTATCAAGAACTTAAAAAATACCTCAAC-3'
RM.CjeNIII rev	5'-GTTGGATCCTATTATATAGTTTTTAGCAAAGTTATATATTCACTTTGCTT-3'
M+S2.VbrI for	5’-GTTCTGCAGTTAAGGTTAATCATATGCCACATTCAAAAATAGATCAAAATGAAATC-3’
M+S2.VbrI rev	5’-TTATTAGGATCCGTCATTAAGCTTGCTTATGATTGTACGGTTTCTGCGTCA-3’
M.VbrORFCP for	5’-GTTCTGCAGTTAAGGTTAATCATATGGCCAAAAACAGCATGAGAAAACA-3’
M.VBrORFCP rev	5’-TTATTAGGATCCTTATTACTGCTCTCTTATAAATAGTTGAAATC-3’
M+S.VbrII for	5’-GTTCTGCAGTTAAGGTTAATCATATGCTGATATCAGGGCTGAAAATGAC-3’
M+S.VbrII rev	5’-TTATTAGGATCCTTATGGAGTACTAGTCATCCTTGTACCTCTTCAGACTTC-3’
M1BceSIII for	5’-TTAGTTGCTGCAGTTAAGGTTAACATATGTTTAAAGAAGAATTAGCTATTGAGAAAATG-3’
M1BceSIII rev top	5’-GCGGGATCCCGTACGGCTTAAGTAGACTTTTTTAAAACAAATACAAAATCTTTATTT-3’
M1BceSIII rev bot	5’-GCGGGATCCACGGCGCCTTAAGTAGACTTTTTTAAAACAAATACAAAATCTTTATTT-3’
M2BceSIII for	5’-TTAGTTGCTGCAGTTAAGGTTAACATATGATAGATTATAATGGTATTGAAAAAAGCTTT-3’
M2BceSIII rev top	5’-GCGGGATCCCGTACGGCCTAATTCTTCTTTAAACATATAACACTTTCCC-3’
M2BceSIII rev bot	5’-GCGGGATCCACGGCGCCCTAATTCTTCTTTAAACATATAACACTTTCCC-3’
CJJ81176_0240 for	5’-AAACTGCAGTTAAGGTGGACATATGAAAGAAAATCCTTCTTTTTTAAAAGAGCAAA-3’
CJJ81176_0240 rev	5’-AAAGGATCCTTAATTTTTAACTAAAATATAAAGTTGTTCATGAAGATGG-3’
CJJ81176_0068 for	5’-AAACTGCAGTTAAGGTGGACATATGAAATTTGAAGCTATAAATGAAAAAGAATTTTT-3’
CJJ81176_0068 rev	5’-AAAGGATCCGCATGCCCTTATTTTCCTTCTATGATTTTTATTTCTTCTTCGGTG-3’
CJJ81176_0713 for	5’-AAACTGCAGTTAAGGTGGACATATGAATTACTATCAAGAACTTAAAAAATACCTCAAC-3’
CJJ81176_0713 rev	5’-AAAGGATCCGCAAGGCCTTGCTTATATAGTTTTTATCAAGGTTATATATTCACTTTG-3’
pRRS srbs	5’-CATATGTTAAACCTTAACTGCAGGCATGCAAGCTTGGC-3’
pRRS rev	5’-TAAGGATCCCCGGGGAAGATCTAGA-3’

[a] Restriction sites used for cloning are underlined, and sequences used for diagnostic purposes are double underlined. The MTase recognition sites are highlighted.

Supplementary Table 2. Summary for all RM system components.

**Bioinformatic predictions**				**Experimental results**	
**ORF #**	**Type**	**Gene**	**Prediction**	**Name**	**Rec. Seq.**[$]
Geobacter metallireducens GS-15					
Gmet_0255	Type II	M (5)	?	inactive	
Gmet_3138	Type II	R	GGATC	GmeIP	GGATC
Gmet_3140	Type II	M	GGATC	M.GmeI	GG^{m6}A$\underline{T}$C
Gmet_0675	Type III	R	?	GmeIIP	(TCCAGG)
Gmet_0676	Type III	M	?	M.GmeII	TCC^{m6}AGG
Chromohalobacter salexigens					
Csal_0084	Type I	M	?	M.CsaII	(CC^{m6}ACN$_6$C$\underline{T}$C)
Csal_0086	Type I	S	?	S.CsaII	CCACN$_6$CTC
Csal_0087	Type I	R	?	CsaIIP	(CCACN$_6$CTC)
Csal_1368	Type II	M	GATC	M.CsaI	RG^{m6}A$\underline{T}$CY
Csal_1401	Type II	M	?	M.CsaIII	^{m6}AB + S^{m6}AAM
Vibrio breoganii 1C-10					
ORF_46A	Type I	S	?	S1.VbrIP	?
ORF_47A	Type I	R	?	VbrIP	(AGHAN$_7$TGAC)
ORF_49A	Type I	S	?	S2.VbrI	AGHAN$_7$TGAC
ORF_51A	Type I	M	?	M.VbrI	(AGH^{m6}AN$_7$$\underline{T}$GAC)
ORF_9B	Type I	M	?	M.VbrII	(CT^{m6}AGN$_6$R$\underline{T}$AA)
ORF_10B	Type I	S	?	S.VbrII	CTAGN$_6$RTAA
ORF_12B	Type I	R	?	VbrIIP	(CTAGN$_6$RTAA)
ORF_50B	Type II	M	GATC	M.VbrIII	G^{m6}ATC
ORF_5C	Type II	M	?	inactive	
Campylobacter jejuni 81-176					
CJJ81176_0776	Type I	M	?	M.CjeFII	(CA^{m6}AYN$_6$AC$\underline{T)}$
CJJ81176_0777	Type I	S	?	S.CjeFII	CAAYN$_6$ACT
CJJ81176_0780	Type I	R	?	CjeFIIP	(CAAYN$_6$ACT)
CJJ81176_1534	Type I	R	?	CjeFIVP	(TAAYN$_5$TGC)
CJJ81176_1536	Type I	S	?	S.CjeFIV	TAAYN$_5$TGC
CJJ81176_1539	Type I	M	?	M.CjeFIV	(TA^{m6}AYN$_5$$\underline{T}$GC)
CJJ81176_0068	Type II	RM	?	RM.CjeFV	GGRC^{m6}A
CJJ81176_0240	Type II	M	GAATTC	M.CjeFI	RA^{m6}A$\underline{T}$TY
CJJ81176_0713	Type II	RM	?	RM.CjeFIII	GCA^{m6}AGG
(CJJ81176_1454	Type II	M	GATC)#		

Campylobacter jejuni NCTC 11168

Cj1549c	Type I	R	?	CjeNIV	(TAAYN$_5$TGC)
Cj1551c	Type I	S	?	S.CjeNIV	TAAYN$_5$TGC
Cj1553c	Type I	M	?	M.CjeNIV	(TA^{m6}AYN$_5\underline{\text{T}}$GC)
Cj0031	Type II	RM*	?		
Cj0208	Type II	M	GAATTC	M.CjeNI	RA^{m6}A$\underline{\text{T}}$TY
Cj0690c	Type II	RM	?	RM.CjeNIII	GKA^{m6}AYG
Cj1051c	Type II	RM	GAGN$_5$GT	RM.CjeNII	G^{m6}AGN$_5$G$\underline{\text{T}}$
(Cj1461	Type II	M	GATC)#		

Bacillus cereus ATCC 10987

BCE_0839	Type I	M	?	M1.BceSVIP	?
BCE_0840	Type I	R	?	BceSVIP	(TAAGN$_7$TTG)
BCE_0841	Type I	M	?	M2.BceSVIP	(TA^{m6}AGN$_7\underline{\text{T}}$TG)
BCE_0842	Type I	S	?	S.BceSVIP	TAAGN$_7$TTG
BCE_0365	Type II	M (5)	GCAGC	M.BceSIV	N/D
BCE_0368	Type II	R	GCAGC	R1.BceSIV	
BCE_0369	Type II	R	GCAGC	R2.BceSIV	
BCE_0392	Type II	M	?	M.BceSVII	promiscuous ^{m6}A
BCE_0393	Type II	M (5)	many%	M.BceSV	N/D
BCE_4604	Type II	R	GGWCC		
BCE_4605	Type II	M (5)	GGWCC	M.BceSII	N/D
BCE_5605	Type II	R	ACGGC	BceSIII	
BCE_5606	Type II	M	ACGGC	M1.BceSIII	A^{m4}CGGC
BCE_5607	Type II	M	ACGGC	M2.BceSIII	G^{m4}CCGT
BCE_1018	Type III	M	CGA^{m6}AG	M.BceSI	CGA^{m6}AG
BCE_1019	Type III	R	CGAAG	BceSI	(CGAAG)

$ recognition sequences for M and R subunits of Type I and the R subunit of Type III enzymes are in parentheses to indicate that they are the effective recognition sequences only. The actual recognition is provided by the S subunit.

* frameshift present

\# possible RNA MTases

% 5'-GGCC-3'/5'-GCNGC-3'/5'-CCGG-3'/5'-GGNCC-3' are all recognized

N/D = not detected

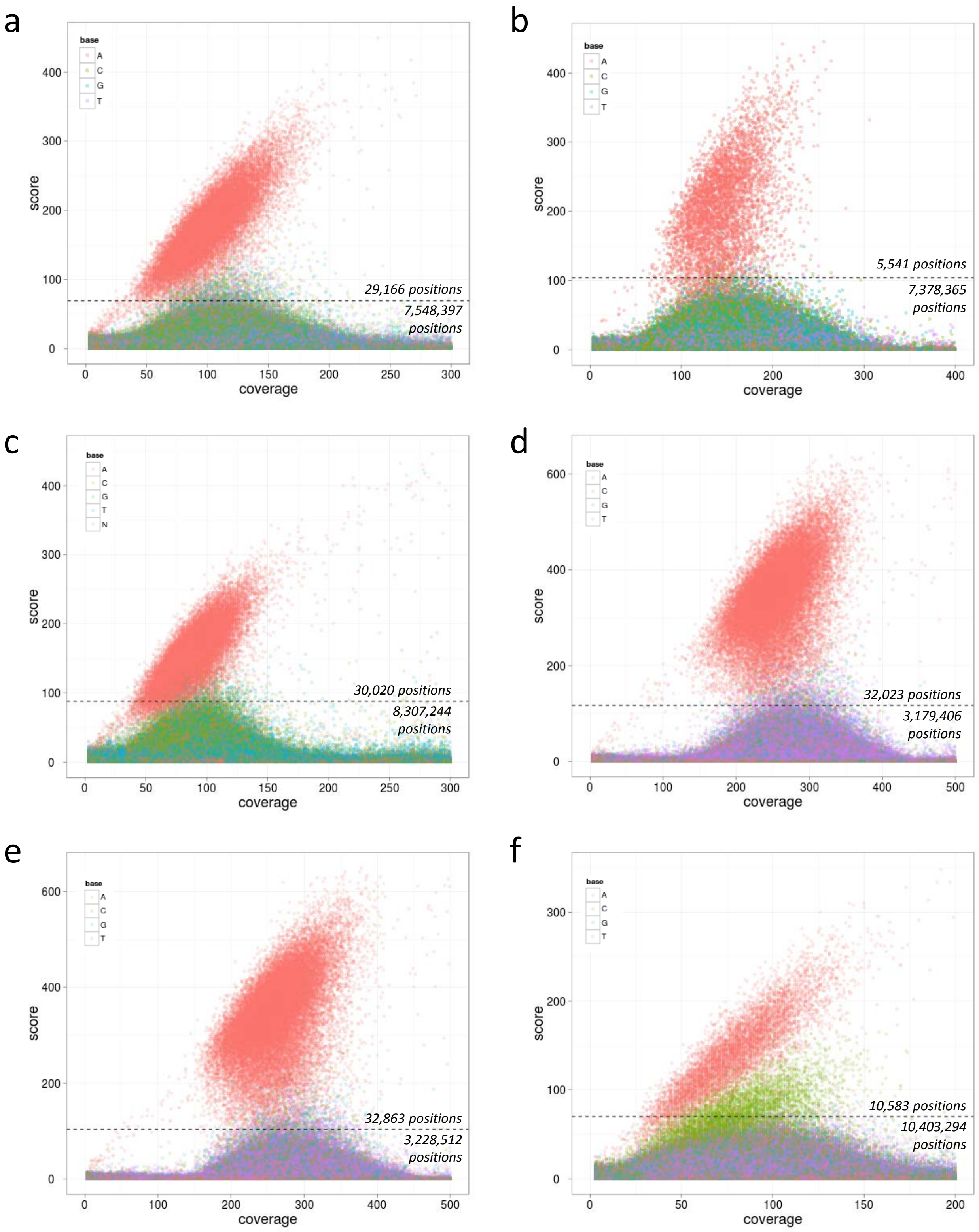

Supplementary Figure 1

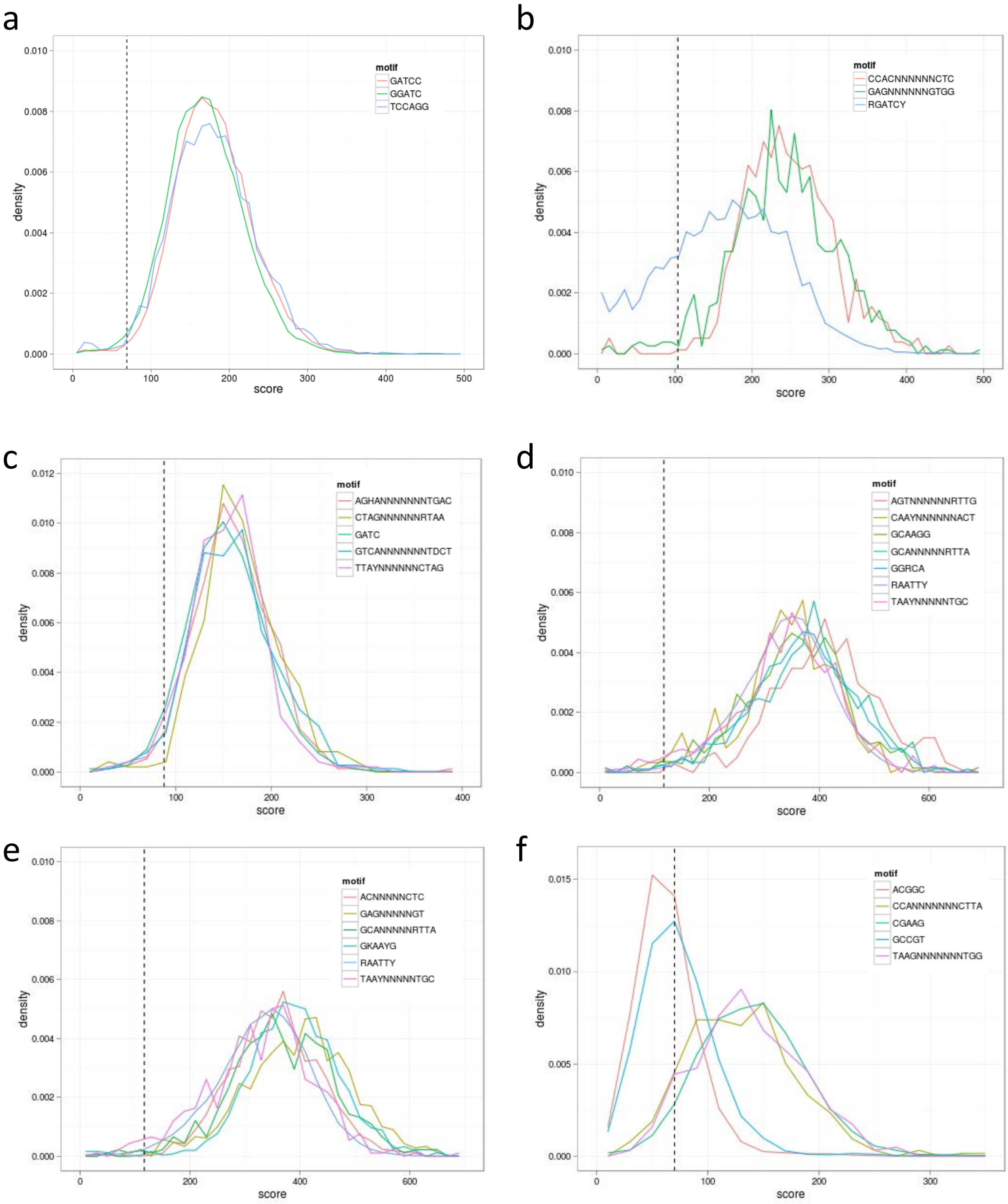

Supplementary Figure 2

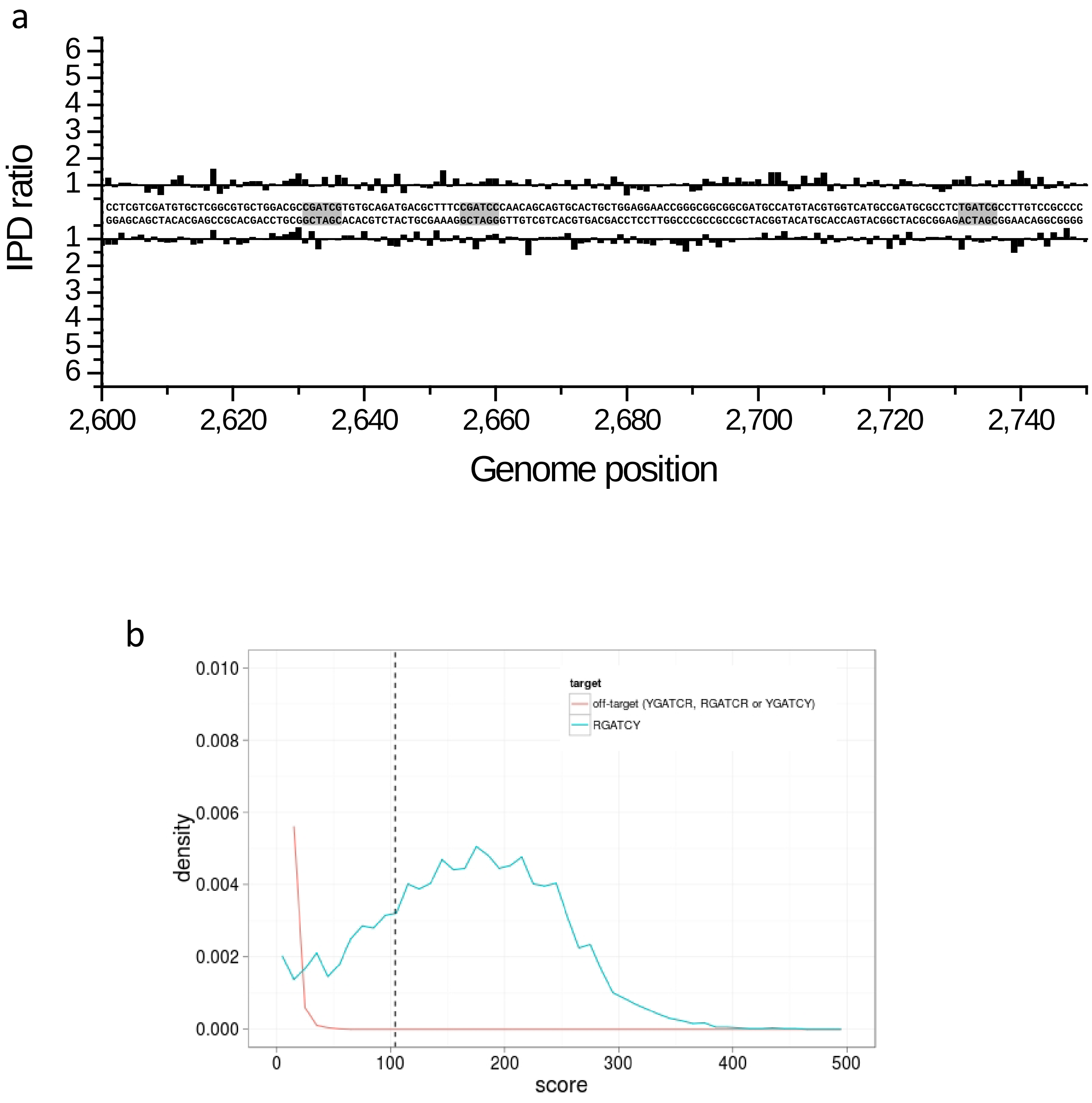

Supplementary Figure 3

a

b

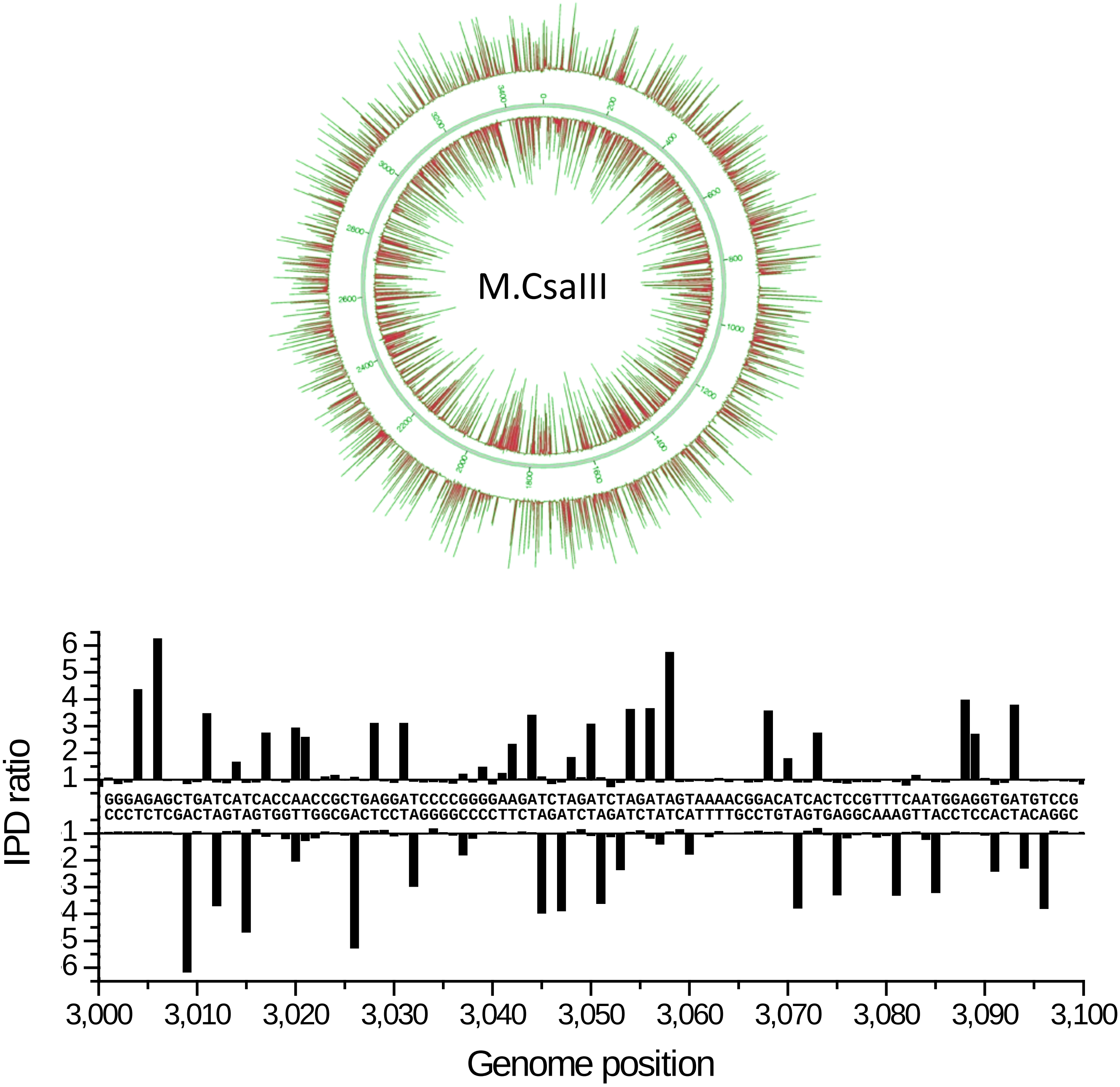

Supplementary Figure 4

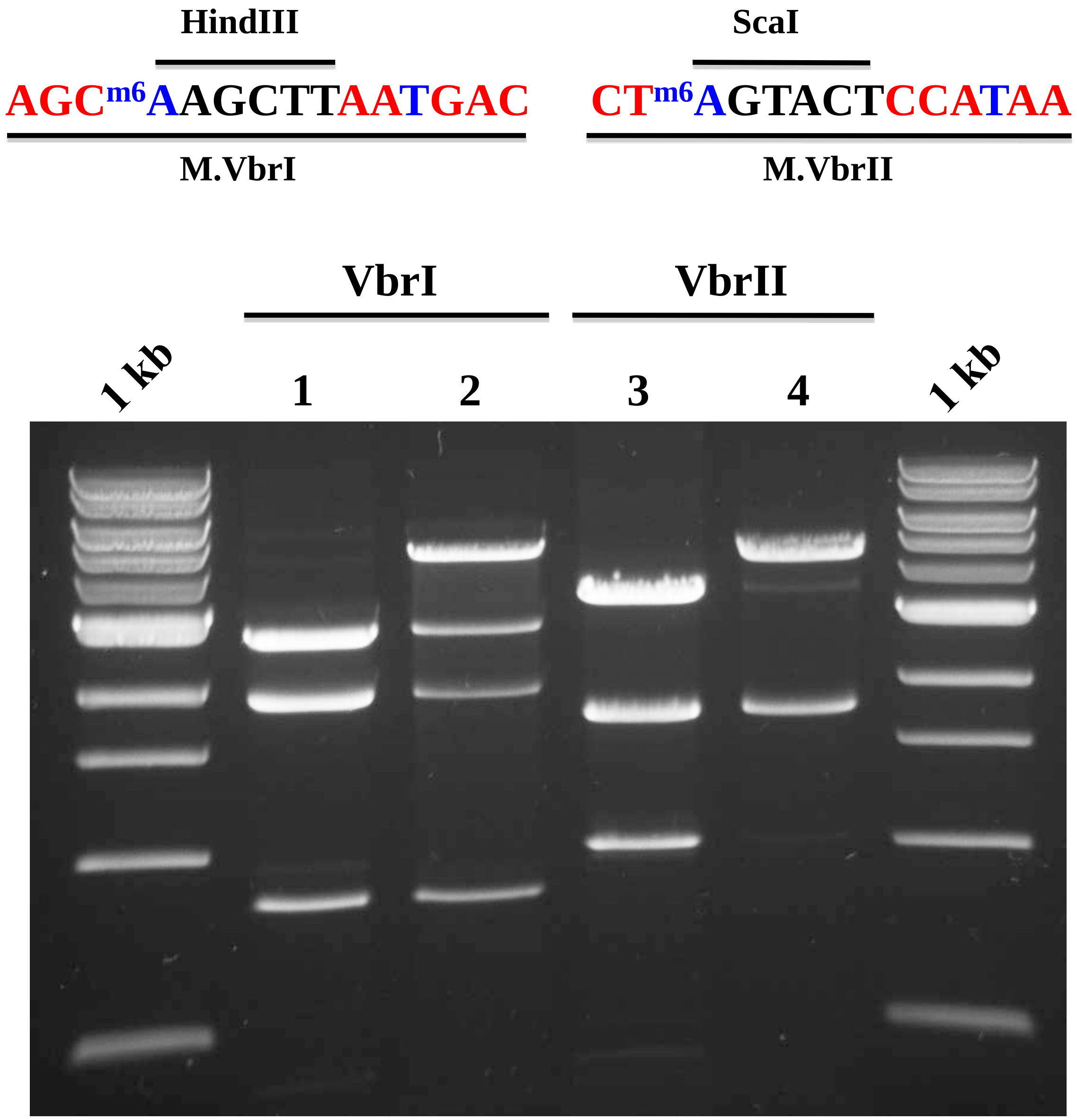

Supplementary Figure 5

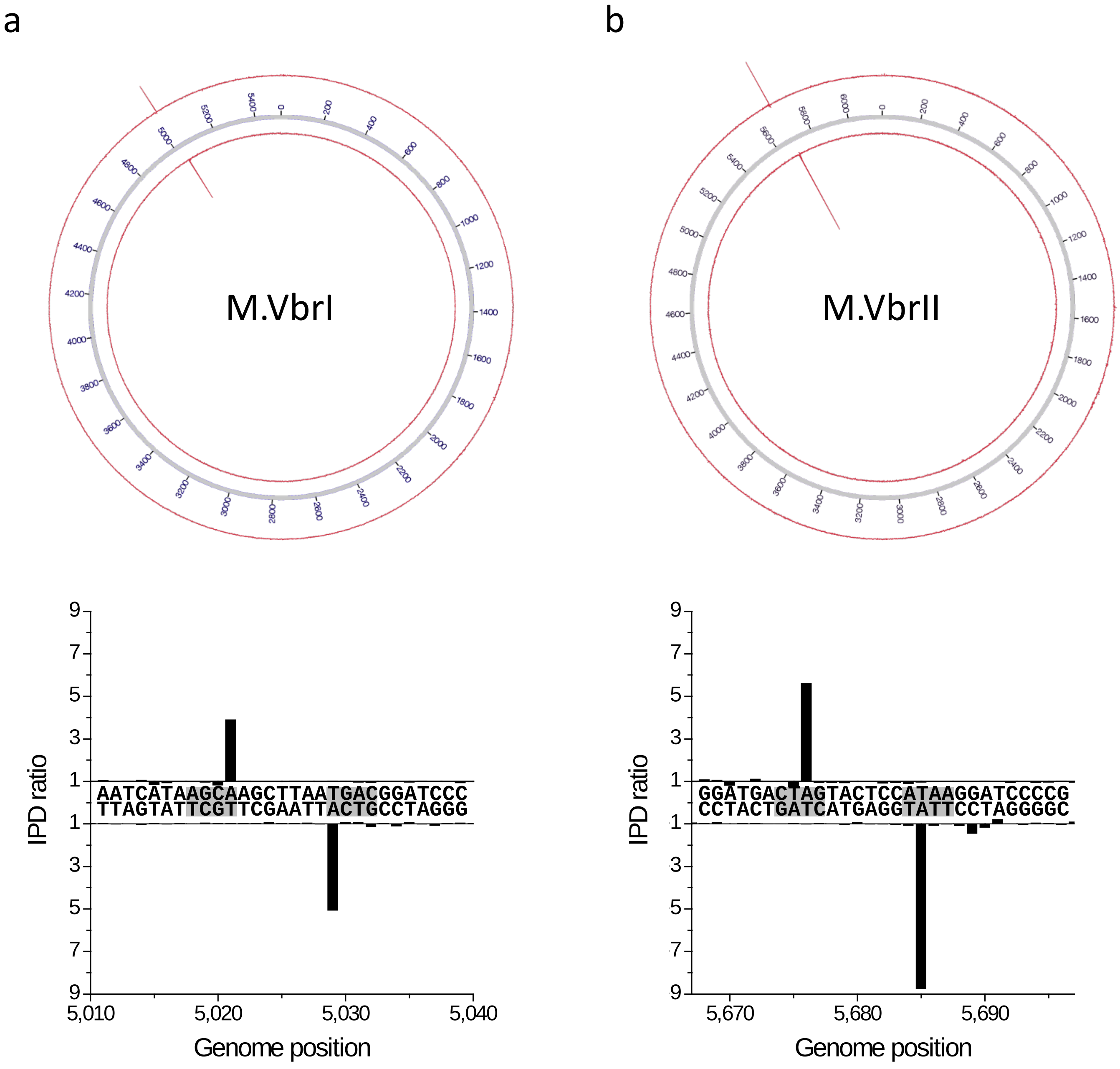

Supplementary Figure 6

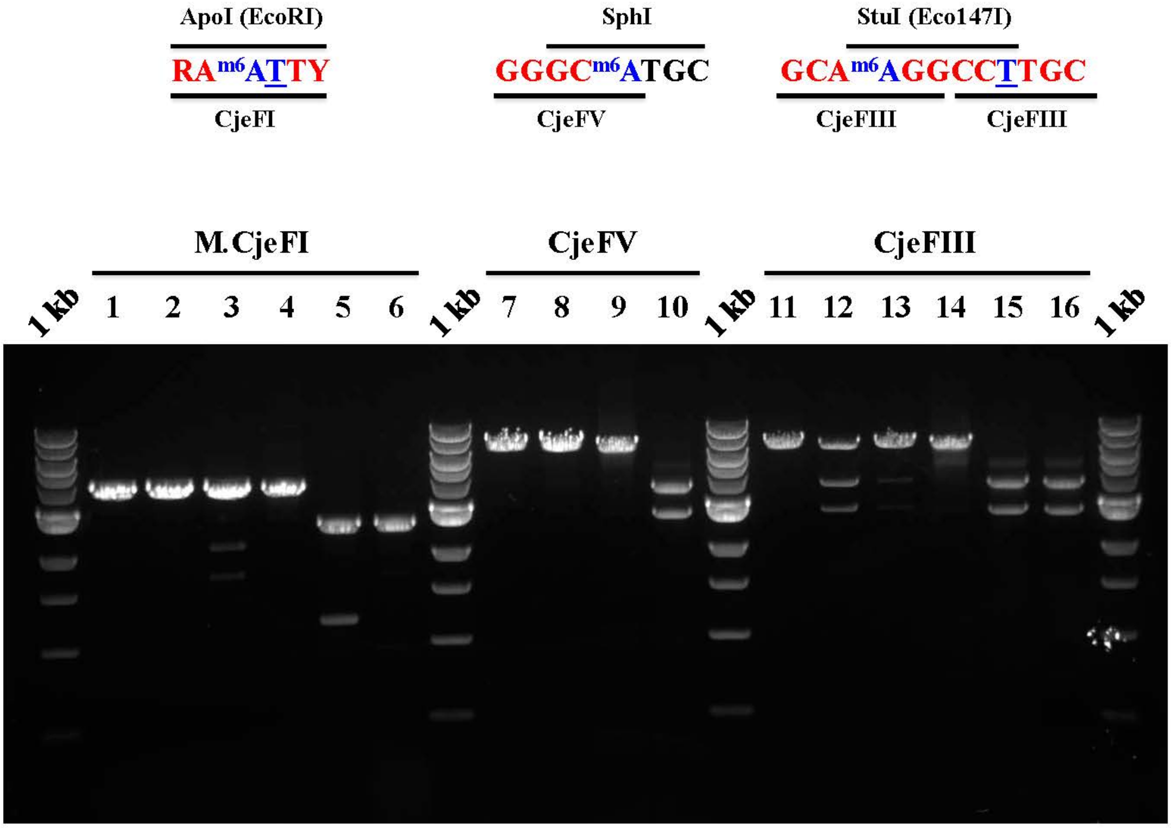

Supplementary Figure 7

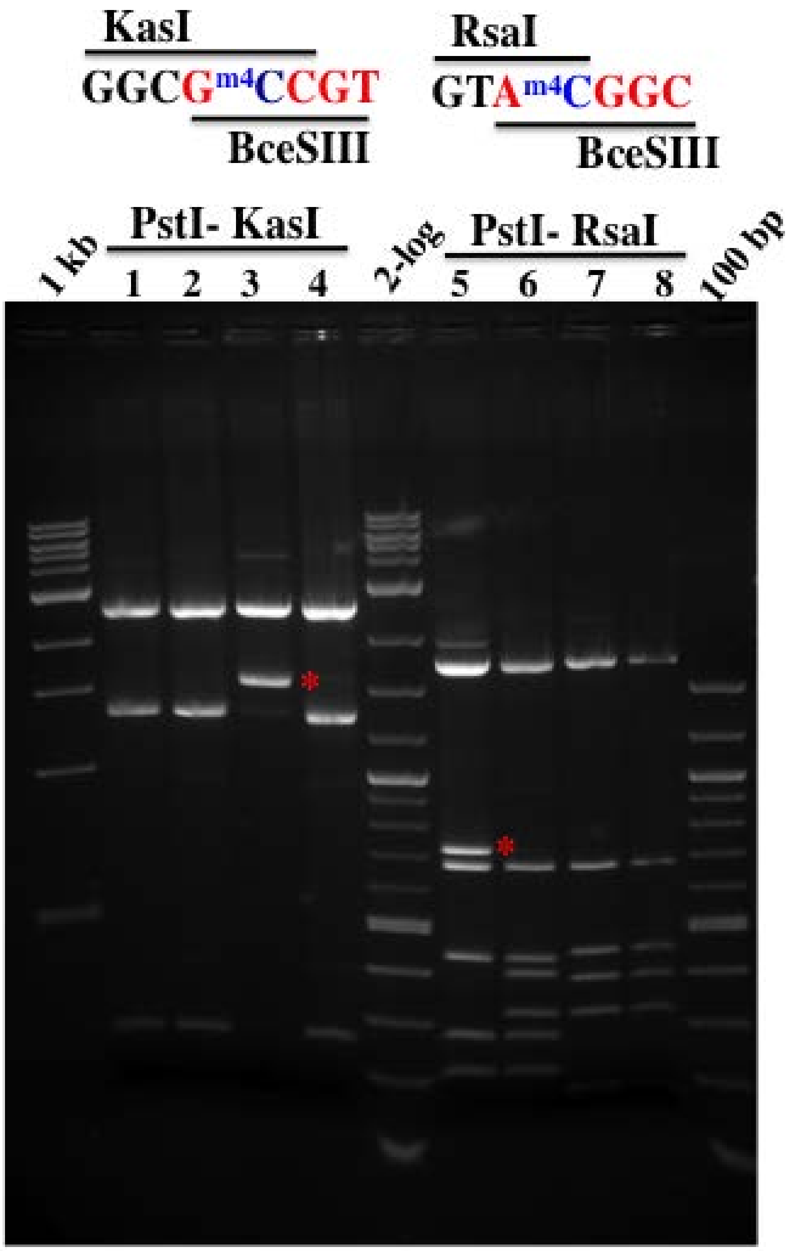

Supplementary Figure 8

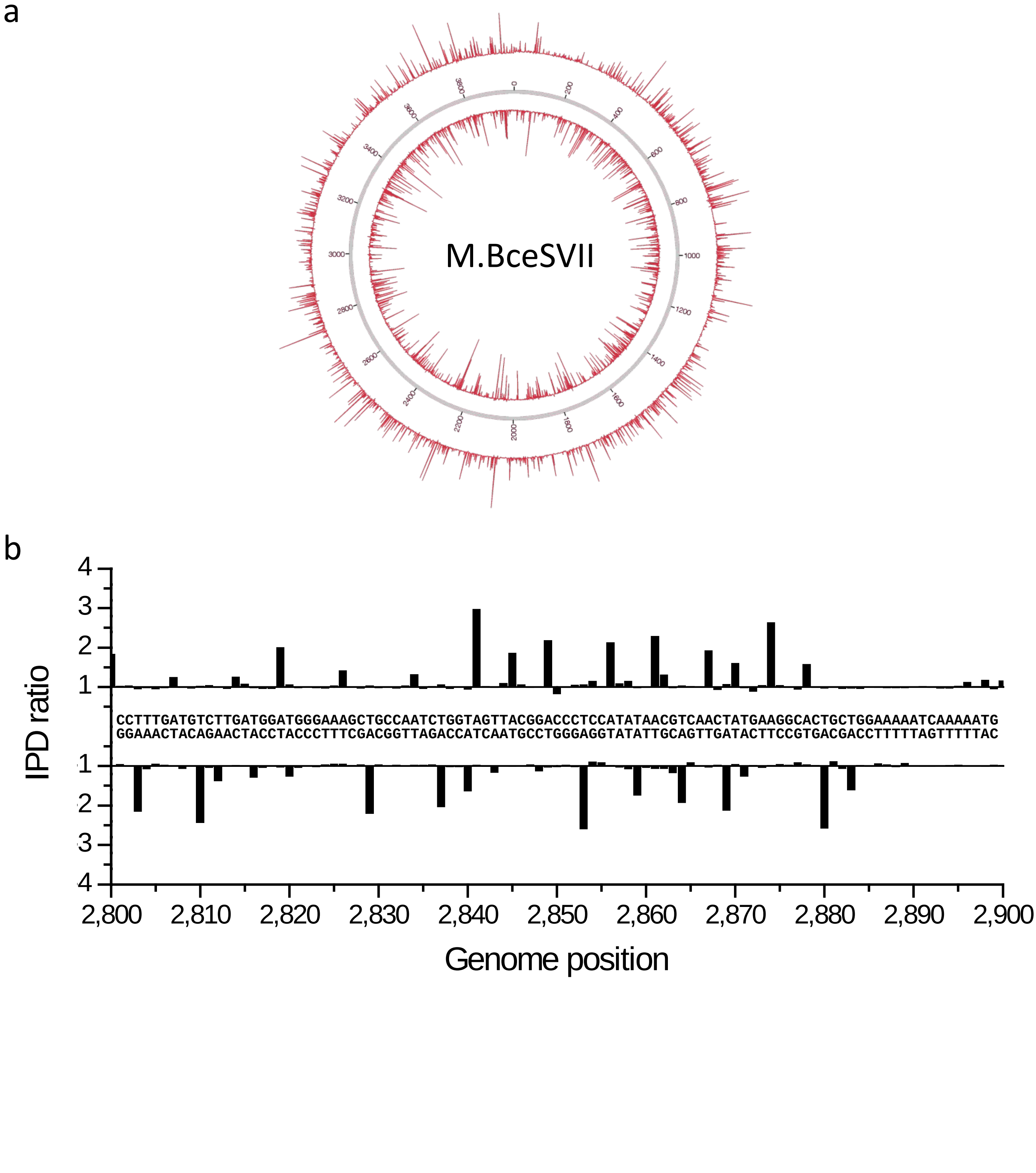

Supplementary Figure 9
